# Plants, pollinators and their interactions under global ecological change: The role of pollen DNA metabarcoding

**DOI:** 10.1111/mec.16689

**Published:** 2022-09-26

**Authors:** Karen L. Bell, Katherine J. Turo, Abigail Lowe, Kevin Nota, Alexander Keller, Francisco Encinas‐Viso, Laura Parducci, Rodney T. Richardson, Richard M. Leggett, Berry J. Brosi, Kevin S. Burgess, Yoshihisa Suyama, Natasha de Vere

**Affiliations:** ^1^ CSIRO Health & Biosecurity and CSIRO Land & Water Floreat WA Australia; ^2^ School of Biological Sciences University of Western Australia Crawley WA Australia; ^3^ Department of Ecology, Evolution, and Natural Resources Rutgers University New Brunswick New Jersey USA; ^4^ National Botanic Garden of Wales Llanarthne UK; ^5^ Department of Ecology and Genetics Evolutionary Biology Centre, Uppsala University Uppsala Sweden; ^6^ Organismic and Cellular Networks, Faculty of Biology Biocenter, Ludwig‐Maximilians‐Universität München Planegg Germany; ^7^ Centre for Australian National Biodiversity Research CSIRO Black Mountain Australian Capital Territory Australia; ^8^ Department of Environmental Biology Sapienza University of Rome Rome Italy; ^9^ Appalachian Laboratory University of Maryland Center for Environmental Science Frostburg Maryland USA; ^10^ Earlham Institute Norwich Research Park, Norwich Norfolk UK; ^11^ Department of Biology University of Washington Seattle Washington USA; ^12^ Department of Biology College of Letters and Sciences, Columbus State University, University System of Georgia Atlanta Georgia USA; ^13^ Field Science Center Graduate School of Agricultural Science, Tohoku University Osaki Miyagi Japan; ^14^ Natural History Museum of Denmark University of Copenhagen Copenhagen Denmark

**Keywords:** DNA metabarcoding, ecosystem change, environmental DNA, global change ecology, metagenomics, pollen, pollination

## Abstract

Anthropogenic activities are triggering global changes in the environment, causing entire communities of plants, pollinators and their interactions to restructure, and ultimately leading to species declines. To understand the mechanisms behind community shifts and declines, as well as monitoring and managing impacts, a global effort must be made to characterize plant–pollinator communities in detail, across different habitat types, latitudes, elevations, and levels and types of disturbances. Generating data of this scale will only be feasible with rapid, high‐throughput methods. Pollen DNA metabarcoding provides advantages in throughput, efficiency and taxonomic resolution over traditional methods, such as microscopic pollen identification and visual observation of plant–pollinator interactions. This makes it ideal for understanding complex ecological networks and their responses to change. Pollen DNA metabarcoding is currently being applied to assess plant–pollinator interactions, survey ecosystem change and model the spatiotemporal distribution of allergenic pollen. Where samples are available from past collections, pollen DNA metabarcoding has been used to compare contemporary and past ecosystems. New avenues of research are possible with the expansion of pollen DNA metabarcoding to intraspecific identification, analysis of DNA in ancient pollen samples, and increased use of museum and herbarium specimens. Ongoing developments in sequencing technologies can accelerate progress towards these goals. Global ecological change is happening rapidly, and we anticipate that high‐throughput methods such as pollen DNA metabarcoding are critical for understanding the evolutionary and ecological processes that support biodiversity, and predicting and responding to the impacts of change.

## 1 INTRODUCTION

Anthropogenic activities are leading to global changes in the environment, including habitat loss (Ellis et al., 2010), climate change (Hansen et al., 2010), biodiversity decline (e.g., Bowler et al., 2020; Butchart et al., 2010), and the spread of invasive species and diseases (Hulme, 2009; Pyšek et al., 2020). Such global changes can act additively or interactively (Didham et al., 2007; Peters et al., 2019) to alter species composition through events such as local introductions and extinctions (Mathiasson & Rehan, 2020; Portman et al., 2018), shifts in phenology (Bartomeus et al., 2011; Forrest, 2015), and changes in the dispersal and connectivity of populations (Damschen et al., 2019). These impacts can subsequently affect the spatiotemporal overlap of species and their behaviour, which can alter species interactions, restructure food webs (Dunn et al., 2018; Kortsch et al., 2015; Richardson et al., 2021) and create network instability (Brosi & Briggs, 2013; Revilla et al., 2015). The negative influence of global change is particularly apparent for plants and their pollinators, where shifts in community composition and function can impair ecosystem services such as pollination (Burkle et al., 2013; Potts et al., 2010). Reduced ecosystem functioning threatens pollinator‐dependent crops, which is likely to impact economic productivity (Reilly et al., 2020) and human nutrition (Smith et al., 2015). Likewise, the changing distribution and phenology of plants with allergenic pollen could challenge human health and quality of life (Anderegg et al., 2021).

With greater understanding of how plant–pollinator interactions vary across space and time, ecologists can better predict how these communities will be affected by global change (Burkle & Alarcón, 2011). More detailed characterization of existing plant and pollinator communities will enable conservation managers to monitor for change, direct management efforts and to iteratively update management processes. To date, there are a handful of ecosystems, mostly in Europe, that have been well characterized in terms of network structure, specialization, and annual turnover in species and interactions. In these study systems, plant–pollinator networks are often nested (i.e., having groups of highly connected species occurring as subsets within larger networks) and modular (i.e., having groups of species, or modules, that have more connections within than between groups) (Olesen et al., 2007). Both nestedness and modularity have been hypothesized to make networks more stable to species loss (Bastolla et al., 2009; Olesen et al., 2007), but networks are dynamic within and between seasons (Olesen et al., 2008). For instance, multiyear studies in Greece have shown that the same pollinator species can interact with a completely different number and identity of plants in different years (Petanidou et al., 2008). These studies imply that networks should be assessed at long temporal scales to account for network dynamism. However, it is unknown whether temporal variation is universal or only apparent in the European study system, as very few long‐term studies have been conducted elsewhere. It is also unknown how plant–pollinator networks are changing with anthropogenic disturbances. To fully understand these processes, detailed, long‐term studies must be conducted across the globe, in different habitat types, latitudes, elevations and levels of disturbance. To collect such a tremendous amount of data on plant–pollinator communities, high‐throughput methods are necessary.

Molecular methods can be useful to quickly obtain large volumes of data on species assemblages, trophic interactions and networks. By combining DNA barcoding with high‐throughput sequencing (HTS), DNA metabarcoding can detect interactions between species (Roslin & Majaneva, 2016) or identify whole communities from environmental samples (Cristescu, 2014). Likewise, complex ecological networks can be efficiently derived from DNA metabarcoding data, because multiple interactions can be detected from a single sample, and taxonomic identification can be obtained for cryptic species and developmental stages or parts of organisms that do not present diagnostic characters (Roslin & Majaneva, 2016). For example, Clare et al. (2019) used DNA metabarcoding of bat faeces in combination with Sanger sequencing of individuals to construct networks of networks containing 3304 interactions between 762 nodes of eight trophic functions involving parasitic, mutualistic and predatory interactions. This scale of information would require years of observation data (Clare et al., 2019), but the authors were able to create complex ecological networks, including cryptic species with molecular data. They were then able to show that bat–prey interactions are much more generalist than bat–parasite and bat–plant networks in their study system, and to identify keystone species based on the number of network connections. Similar results collected across biomes could greatly improve rapid monitoring of global plant–pollinator communities and contribute to the construction of long‐term data sets.

**FIGURE 1:**
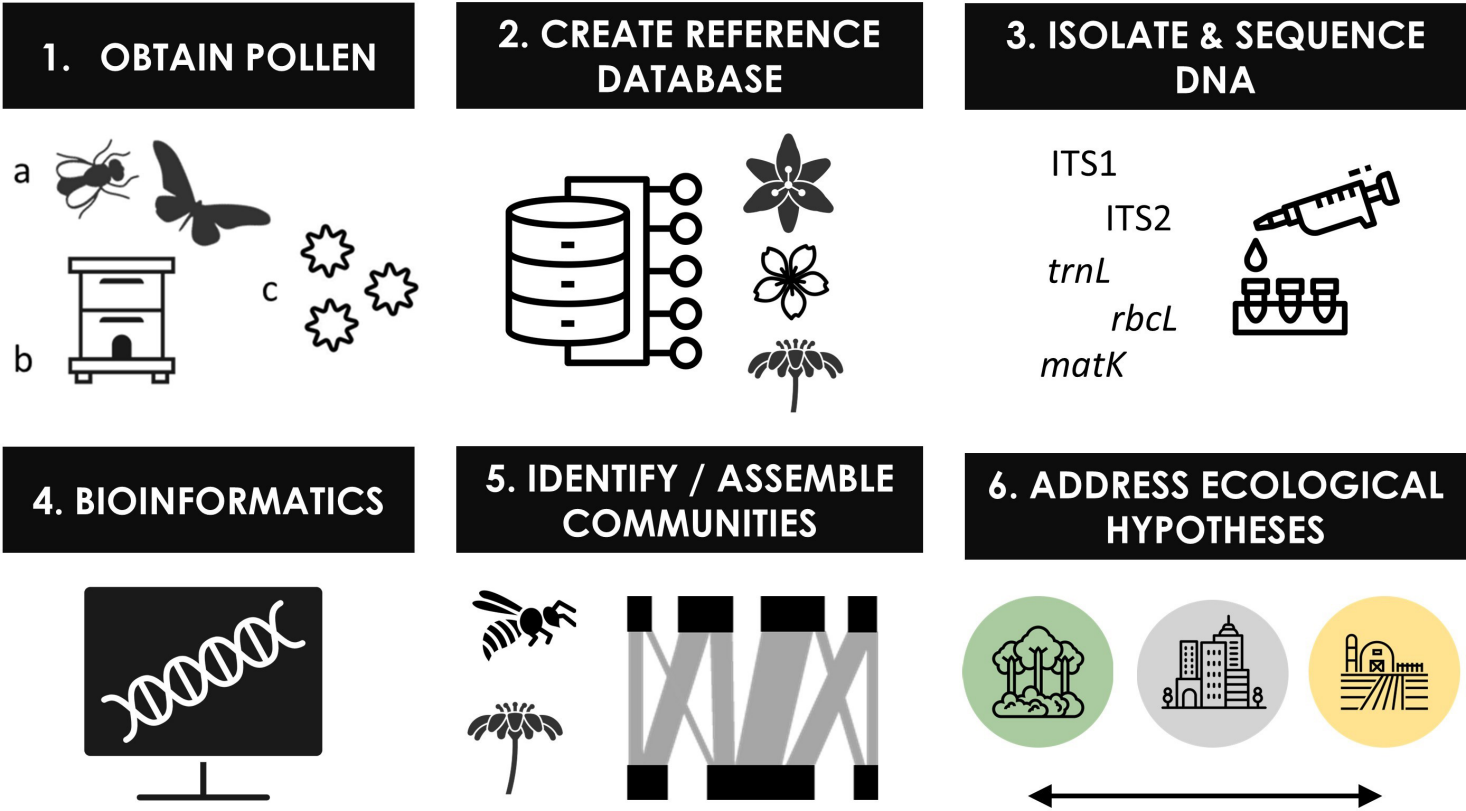
Research workflow when using DNA‐based pollen identification. Methods will vary depending on the ecological question of interest, the study system, the availability of existing reference sequences and funding. Much of the contemporary research on pollen metabarcoding has focused on methods development (Figure S1), but recent applications in global change ecology have risen over the past 2 years (Table S1), highlighting pollen metabarcoding's capacity for addressing ecological hypotheses.

Pollen is a powerful biomarker for detecting spatial and temporal variation in plant and pollinator species assemblages and interactions, making it ideal for high‐throughput assessment of global ecological change (Hornick et al., 2021). Traditionally, taxonomic identification of pollen is based on the visual observation of pollen morphology, but is limited in throughput (Stillman & Flenley, 1996) and taxonomic resolution (Lau et al., 2019; Mander & Punyasena, 2014; Richardson et al., 2019). Research identifying species or genotypes of plants using DNA from pollen began in the 1990s and was based on genotyping of individual pollen grains (Petersen et al., 1996; Suyama et al., 1996). With the advent of HTS technology, DNA‐based pollen identification is no longer dependent on the time‐consuming isolation and analysis of DNA from individual pollen grains (Aziz & Sauve, 2008; Matsuki et al., 2007). Instead, with HTS, researchers have been able to sequence pollen from bulk samples using DNA metabarcoding, following procedures available in most molecular biology laboratories (Figure 1). This breakthrough has allowed rapid, large‐scale identification of species within mixtures. Early proof‐of‐concept papers on pollen DNA metabarcoding demonstrated the feasibility of the method (e.g., Cornman et al., 2015; Hawkins et al., 2015; Keller et al., 2015; Kraaijeveld et al., 2015; Richardson et al., 2015) and it has since been used in a range of applications (Appendix S1: Methods S1, Figure S1, Table S2; Appendix S2).

Pollen DNA metabarcoding provides an important tool for understanding and monitoring ecosystems under global change. Here, we review current knowledge on the impacts of global change on plants, pollinators and their interactions, and the knowledge gaps that pollen DNA metabarcoding is well placed to address (Section 2); provide an assessment of progress on technical issues that may be preventing the application of pollen DNA metabarcoding to research questions (Section 3); and envisage additional concepts that could be addressed with methods development and incorporation of new and emerging technologies (Section 4).

## 2 CONTEMPORARY APPLICATIONS OF EXISTING POLLEN DNA METABARCODING METHODS IN GLOBAL CHANGE ECOLOGY

**FIGURE 2:**
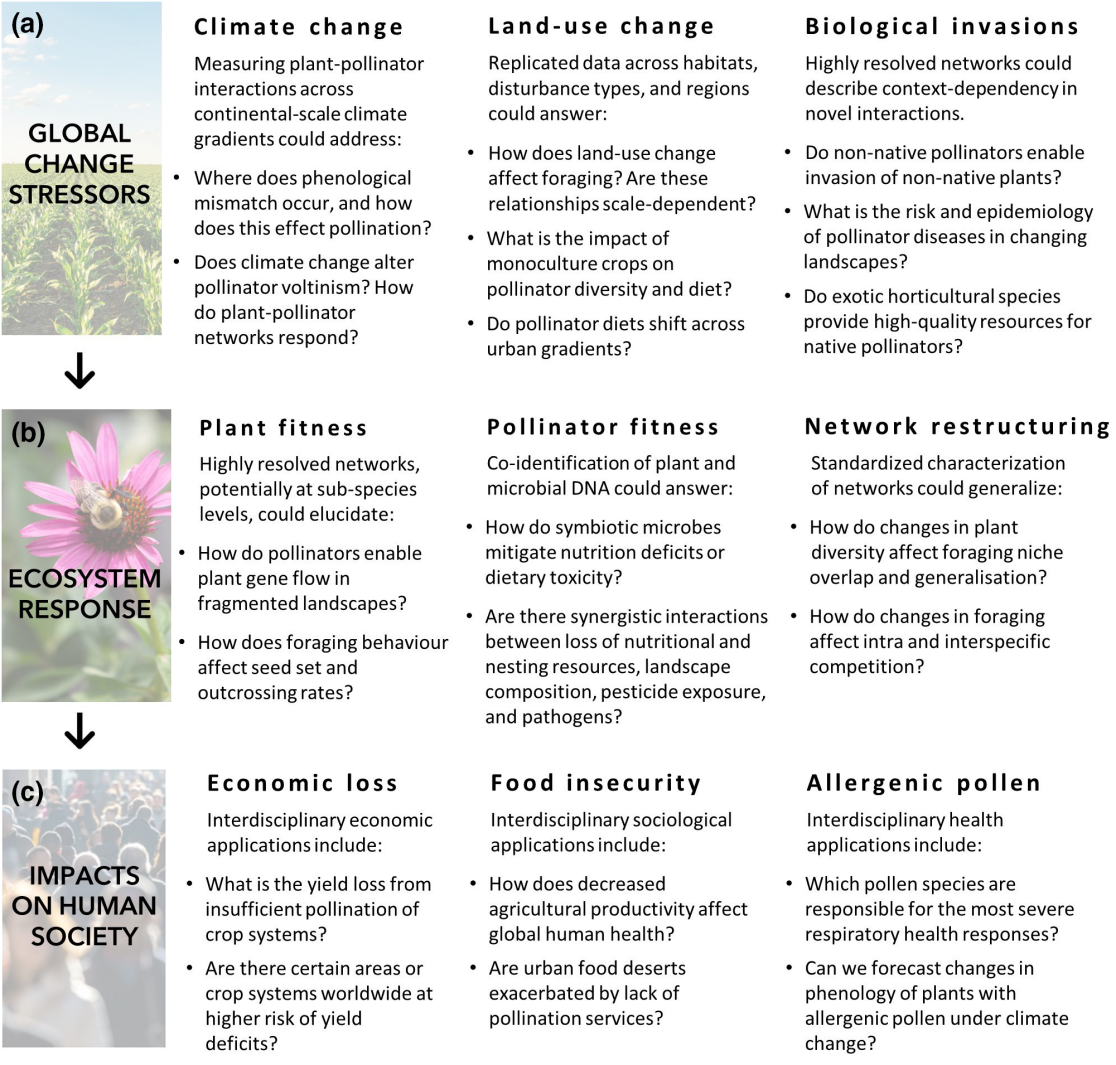
Many perennial questions in global change ecology are constrained by technical difficulties in identifying plant–pollinator interactions or environmental pollen at global scales. The major advantage of pollen DNA metabarcoding is improved detection and quantification of plant species from pollen samples. DNA methods can accommodate increased sampling (higher throughput), more accurately identify plant species and plant–pollinator interactions (higher resolution) and characterize plant–pollinator networks across unique habitats and gradients (e.g., climate, land‐use) for regional and global comparisons (scalability). Thus, we highlight three research themes and affiliated research questions where pollen metabarcoding could be applied to help society continue to understand, anticipate and adapt to global ecological change.

Traditional observation‐based plant–pollinator interaction research and network theory have generated predictions of how species and their interactions might respond to environmental changes (Bascompte & Jordano, 2007; Burkle & Alarcón, 2011; Revilla et al., 2015). Plant–pollinator interaction networks are predicted to be quite plastic, with many species persisting following disturbance. Network stability can be maintained after a disturbance through changes in mutualistic partners until a certain threshold is reached, where the ecosystem collapses (Fortuna & Bascompte, 2006). Network responses are expected to be heterogeneous with respect to time and space, and the impacts of specific changes such as habitat loss, climate change and biological invasions are poorly understood (Burkle & Alarcón, 2011). These hypotheses have been tested at local scales using traditional methods, and results have been consistent with the expectation that many interactions are lost but the overall network structure is stable (e.g., Burkle et al., 2013). However, large amounts of data are needed to test whether this expected heterogeneity occurs across geographical ranges and disturbance types. Given the scalability of pollen DNA metabarcoding, this method has the potential to improve our conceptual understanding of plant–pollinator network structure and response to change, including understanding cascading impacts of changing interactions on pollinator health, impacts of land‐use change, impacts of biological invasions and impacts of climate change on plant phenology and diversity (Figure 2). Several recent pollen DNA metabarcoding studies have corroborated the broader theory developed from traditional methods, and addressed specific hypotheses related to management that would not have been practical if relying on morphological identification of pollen by expert palynologists (Gresty et al., 2018). Analysis of pollen provides a further opportunity to improve our understanding of the impacts of ecosystem change on pollinator health, because the same pollen sample can be used for detection of plant–pollinator interactions, analysis of nutritional value (Donkersley et al., 2017), and analysis of microbiome species diversity and composition (Leonhardt et al., 2022). Finally, pollen DNA metabarcoding can be used for high‐throughput biodiversity monitoring of plant community composition (Leontidou et al., 2021; Milla et al., 2022), and the transport and deposition of airborne allergenic pollen in current environments and under different climate change scenarios (Campbell et al., 2020; Rowney et al., 2021; Uetake et al., 2021).

### 2.1 Understanding the impact of ecosystem change through high‐resolution plant–pollinator interaction networks

How does land‐use intensification and urbanization affect bee foraging (Peters et al., 2022)? Which bee species can or cannot cope with such anthropogenic changes (Peters et al., 2022)? How does this in turn affect pollination of plants (Hornick et al., 2021)? How is bee foraging, insect migration and pollination linked to climatic changes (Mayr et al., 2021; Suchan et al., 2019; Figure 2)? Answering such questions requires deep taxonomic resolution of networks and the inclusion of different levels of human impact, at multiple time points, locations and habitat types, each with appropriate replication due to natural variation (Burkle & Alarcón, 2011). Visitation networks can be constructed through a range of methods which that are focused on the perspective of the plant, animal or a combination of both (Lowe et al., 2022). To address the impacts of anthropogenic changes on insect health, the dietary intake of the flower‐visiting animal is required, information which cannot be achieved completely through most plant‐focused surveys (Arstingstall et al., 2021; Parreño et al., 2022; Popic et al., 2013; Ruedenauer et al., 2016). Pollen‐based methods, such as DNA metabarcoding, provide the animal perspective and allow exploration of the dietary input obtained from these interactions (Piko et al., 2021; Pornon et al., 2017; Zhao et al., 2018). Dietary input can be explored at different scales from the preferences of individual foragers (Biella et al., 2019; Casanelles‐Abella et al., 2021; Elliott et al., 2021; Piko et al., 2021) to colony‐, nest‐ or species‐level assessments (Danner et al., 2017; Nürnberger et al., 2019; Sickel et al., 2015). Furthermore, analysing resource partitioning and specialization at different scales, from the community to the individual, improves our understanding of how plant–pollinator relationships are organized and how vulnerable they are to ecosystem change (Brosi, 2016; Elliott et al., 2021; Lucas et al., 2018; Peters et al., 2022). The ability to characterize an individual's entire pollen assemblage makes metabarcoding uniquely placed to explore novel questions on individual specialization at a large scale and is a promising area of future research (Lowe et al., 2022). Using DNA metabarcoding to characterize resource partitioning throughout a network has identified that generalized networks and species are composed of specialized individuals (Lucas et al., 2018; Pornon et al., 2019). Prior to the application of high‐throughput and high‐resolution methods such as metabarcoding, individual‐based networks were seldom explored due to construction being time consuming, owing to the high sampling effort required for visual surveys or the morphological identification of pollen (Tur et al., 2014). Longer‐term sampling and analysis of museum specimens (Gous et al., 2021) can provide comprehensive insights into the complete foraging spectrum and species' dietary niche, co‐evolution and long‐term responses to changes (Kaluza et al., 2017; Vaudo et al., 2020; Wilson et al., 2021).

### 2.2 Relationships between pollinator foraging, nutrition and health

Pollinator health has been affected by anthropogenic change, but the mechanisms behind these impacts are still poorly understood. There are likely to be synergistic interactions between loss of nutritional and nesting resources as well as other factors such as landscape composition, pesticide exposure and pathogens (Parreño et al., 2022; Woodard & Jha, 2017). Holistic approaches that link nutrition, microbial community structure, pathogen, parasite and viral load allow us to understand the complex interactions between the individual factors (López‐Uribe et al., 2020). For example, using pollen DNA metabarcoding coupled with chemical analyses of nutritional composition, several recent studies have shown that plant resource diversity is directly linked to pollinator nutrition, development and health, and reduced resource diversity in intensive agricultural landscapes can negatively impact nutritional profiles (Donkersley et al., 2017; Peters et al., 2022; Trinkl et al., 2020). This information is only traceable with bee‐derived data as produced by DNA metabarcoding. Knowing the pollen composition, we can identify malnutrition risks caused by switching to unfavourable or fewer food sources (Brodschneider & Crailsheim, 2010; Eckhardt et al., 2014; Peters et al., 2022; Ruedenauer et al., 2016; Trinkl et al., 2020; Vanderplanck et al., 2018).

Recently, the importance of transmitting microbes between plants and pollinators by hitchhiking on pollen grains, nectar and animal bodies has been recognized (Keller et al., 2021; McFrederick & Rehan, 2016; Zemenick et al., 2021). This accounts both for microbes with beneficial (Dharampal et al., 2019; Leonhardt et al., 2022; Vuong & McFrederick, 2019) and detrimental (Keller et al., 2018; Voulgari‐Kokota et al., 2020) effects on host ecology, nutrition and health (Engel et al., 2016; Leonhardt et al., 2022; Vannette, 2020; Voulgari‐Kokota et al., 2018). Land‐use changes are known to not only affect the diversity of plants but also lead to reduced diversity and homogenization of microbial communities in flowers (Gaube et al., 2021) and, with less pollination network modularity and resource competition on dominant hubs, increase the risk of pathogen transmission (Keller et al., 2021; Zemenick et al., 2021). Coupled pollen and microbiome DNA metabarcoding has already been applied to understand the role of pollen foraging on microbiome compositions, for example that florally derived microbes largely shape the pollinator microbiomes of the nest, larvae and adults, especially for solitary bees (Keller et al., 2018; Leonhardt et al., 2022; McFrederick & Rehan, 2016, 2019; Voulgari‐Kokota et al., 2019). Microbial genomics coupled with pollen foraging assessments through metabarcoding allow us to understand how microbes influence interaction networks by changing resource attractiveness (Keller et al., 2021; Vannette, 2020) or bee cognitive adaptation capabilities (Zhang et al., 2022), where microbe symbioses might mitigate nutrition deficits (Kwong et al., 2014; Leonhardt et al., 2022; Vuong & McFrederick, 2019) or dietary toxicity (Zheng et al., 2016), and what the risk and epidemiology of pollinator diseases (Castelli et al., 2020) are in changing landscapes (Figure 2).

### 2.3 Pollinator responses to land‐use change

In recent decades, there have been broad‐scale shifts in land use with subsequent changes in resource diversity and availability to pollinators (Burkle et al., 2013; Jones, Brennan, et al., 2021; Scheper et al., 2014). Overall, there has been deforestation in the tropics but widespread reforestation and afforestation in temperate regions, often with monocultures or small numbers of tree species replacing previous flora (Song et al., 2018). In addition, land cover changes from increasing urbanization and afforestation (He et al., 2019; Song et al., 2018) often occur at the expense of grassland and herbaceous areas, which probably contain important pollinator habitat. How do these landscape‐scale changes in resource availability and diversity affect plant–pollinator communities and their interactions? Are there land‐use changes that destabilize or alter plant–pollinator networks?

DNA metabarcoding can detect higher numbers of species than observational data (Arstingstall et al., 2021; Pornon et al., 2017), which is a significant advantage when considering how landscape context influences plant–pollinator interactions. This advantage has been used to assess the extent of seed mix utilization (Gresty et al., 2018; McMinn‐Sauder et al., 2020) and highlighted the need for a landscape perspective of foraging (Piko et al., 2021). Likewise, landscape‐scale resource diversity is frequently important for social pollinators (Kaluza et al., 2017). Social bees often collect a high diversity of plants at low densities (de Vere et al., 2017; McMinn‐Sauder et al., 2020; Wilson et al., 2021), especially when considering foraging at longer timescales (Sponsler, Grozinger, et al., 2020). This is true even in resource‐poor landscapes, where honey bees communicate to maximize pollen collection from diverse plant resources (Nürnberger et al., 2019). Likewise, Danner et al. (2017) showed that honey bees adapt to reduced landscape plant diversity by increasing foraging ranges, and both Tommasi et al. (2021) and Peters et al. (2022) linked increasing urbanization and agricultural land‐use with greater generalization and foraging niche overlap among pollinators. In all these cases, pollen metabarcoding enabled more detailed detection of how pollinators use floral resources at landscape scales. These findings advance our understanding of the role of plant diversity in supporting generalist pollinators and imply that landscape‐scale management is critical for maintenance of plant–pollinator networks.

In addition, pollen DNA metabarcoding has been used to understand the flexibility of pollinator species' dietary niche in response to changing landscapes (Casanelles‐Abella et al., 2021; Vaudo et al., 2020). These studies have confirmed that generalist species switch host plants depending on availability across landscape types (Casanelles‐Abella et al., 2021) while oligolectic and mesolectic bee species exhibit diet conservatism to host‐plant species within their preferred plant families (Casanelles‐Abella et al., 2021; Vaudo et al., 2020). Dietary flexibility enables pollinators to adapt to changing environments and maintain the stability of plant–pollinator networks, but dietary niche breadth remains poorly characterized for most species. Thus, pollen metabarcoding has an important role in evaluating bee diets and understanding the mechanisms underlying ecosystem resilience to land‐use change.

Much of the work in landscape‐scale assessments of pollinator foraging has focused on agroecosystems. Expansions in agricultural land cover can have a range of impacts on pollinators, including decreasing floral resource diversity (da Rocha‐Filho et al., 2021; Jones, Brennan, et al., 2021; Lucek et al., 2019;Richardson et al., 2021; Samuelson et al., 2020), and increasing pesticide and parasite exposure (Cohen et al., 2021; Douglas et al., 2020; Douglas & Tooker, 2015). Pollen DNA metabarcoding can be used to elucidate and monitor how pollinator foraging responds to changing agroecosystems. For instance, a comparison of modern‐day honey with samples collected 65 years previously demonstrated shifts in honeybee forage composition in response to changes in agricultural practices and the distribution of invasive species within the UK, illustrating adaptability in major forage use by honey bees (Jones, Brennan, et al., 2021). Recent studies, using both molecular and morphological pollen identification, have also found that honeybees situated in modern agricultural landscapes tend to collect a lower diversity of forage relative to nearby nonagricultural landscapes (Richardson et al., 2021; Samuelson et al., 2020), and land‐use intensification decreases pollinator richness (Tommasi et al., 2021). As agroecosystems continue to expand and incorporate new management, it is increasingly important to ask: how does agricultural intensification and diversification influence pollinator foraging behaviour and health, and the stability of the pollination services they provide (Figure 2)? To answer this, greater spatial replication and comparisons over a broader diversity of alternative landscape types are needed, two challenges to which molecular pollen identification is uniquely suited.

While both agricultural and urban intensification can negatively impact pollinator communities through habitat loss (Potts et al., 2010), many urban environments also support rich pollinator diversity (Baldock et al., 2015; Hall et al., 2017; Turo & Gardiner, 2019), especially when landscapes exhibit moderate or intermediate levels of urbanization (Wenzel et al., 2020). Improved characterization of urban foraging networks is therefore important for understanding why certain pollinators perform well in cities and for informing the development of urban pollinator habitat, which frequently does not consider pollinator foraging preferences or nutrition (Garbuzov & Ratnieks, 2014). Pollen DNA metabarcoding is ideal for quantifying plant–pollinator interactions and pollinator diets in urban environments. Traditional methods for monitoring plant–pollinator interactions (e.g., flower–visitor observations, hand‐netting) can be challenging to use in urban areas due to restrictions on sampling private property and the high floral diversity present in the urban matrix (Sponsler, Shump, et al., 2020).

Recent metabarcoding studies circumvent these challenges and illustrate how pollinators partition floral resources (especially native and non‐native forage) (Potter et al., 2019; Sponsler, Shump, et al., 2020) within unique urban contexts and across large‐scale urbanization gradients (Casanelles‐Abella et al., 2021). Importantly, these studies found that urban pollinators forage pollen from a wide range of native and introduced plants (e.g., ornamental, weeds) and reveal a highly seasonal structure of floral resources (Potter et al., 2019; Sponsler, Grozinger, et al., 2020); however, they also showed that diet composition of urban pollinators changes depending on the level of urbanization (Casanelles‐Abella et al., 2021). Future application of pollen DNA metabarcoding in urban environments could build off these studies by connecting whether certain diets, for example higher proportions of tree pollen (Casanelles‐Abella et al., 2021) or native forage (Potter et al., 2019), are associated with improved reproductive success. Likewise, genomic data could promote greater understanding of unique urban challenges. For instance, molecular reconstruction of urban pollinator foraging could be used to test whether warming associated with urban heat islands leads to reduced complexity of foraging networks (Hamblin et al., 2018), or if interspecific competition for floral resources by non‐native pollinators depletes the diet of native species (Fitch et al., 2019).

Further opportunity to examine the impacts of land‐use change on pollinators is available through historical samples. Examination of historical bee specimens in the Netherlands and pollen identification by microscopy has shown that the decline of preferred host plants was associated with bee decline (Scheper et al., 2014). Pollen DNA metabarcoding of museum specimens provides a higher throughput and higher resolution method for comparing foraging changes through extended time periods (Gous et al., 2019; Gous et al., 2021; Jones, Brennan, et al., 2021). More recently, pollen DNA metabarcoding of museum specimens has been used to assess the relationship between host plant availability and declines of the endangered *Bombus affinis* in North America (Simanonok et al., 2021). In contrast to earlier research in other study systems, they observed no dramatic changes in pollen composition over time on bee specimens dating back 100 years, illustrating that the *B. affinis* decline is probably not driven by changes in specific floral resources. To clarify how pollinator diet has changed throughout the Anthropocene, researchers should conduct further DNA metabarcoding of pollen from museum specimens across a variety of pollinator taxa and landscapes. Such historical perspectives will elucidate if pollinator declines are connected to host plant availability and how plant–pollinator interactions change in relation to changes to climatic variables, allowing more precise predictions for future climate change scenarios.

### 2.4 Impacts of biological invasions on plant–pollinator interactions

The impacts of biological invasions on plant and pollinator communities are wide‐ranging and variable. The integration of non‐native plants into native plant–pollinator networks requires the attraction of pollinators (native or introduced), successful reproduction and population growth, and an impact on plant–pollinator interaction network structure (Parra‐Tabla & Arceo‐Gomez, 2021). Novel plants may be visited by pollinators that are already present in the network, or may attract new generalist pollinators into the network (Russo et al., 2014). Either strategy increases generalization in the network, which can impact the modularity and nestedness and therefore network stability (Albrecht et al., 2014; Stouffer et al., 2014). While the impacts of non‐native species on plant–pollinator interaction networks have been studied in detail for many study systems, these impacts have been shown to be context‐specific, varying with phylogenetic relatedness and phenotypic matching of native and non‐native neighbours (Gibson et al., 2012; Morales & Traveset, 2009; Vaudo et al., 2020), as well as invasion intensity (Kaiser‐Bunbury et al., 2011). Further experimental research is needed to determine the impacts of non‐native species in plant–pollinator networks and should consider the role of functional traits (Gibson et al., 2012; Johnson & Ashman, 2019), overlap in phenology of native and non‐native plants (Bartomeus et al., 2008), and what threshold of non‐native abundance disrupts plant–pollinator networks (Vilà et al., 2009).

Because the impacts of non‐native species are context‐specific, improving conceptual understanding will require detailed analysis across a diverse range of study systems. Pollen DNA metabarcoding has begun to provide answers to questions on non‐native species and the restructuring of interaction networks, by improving detailed species‐level understandings (Vaudo et al., 2020; Wilson et al., 2021). These studies tend to be focused on foraging behaviour of non‐native pollinators. This has improved conceptual understandings of how pollinators choose their host plants, and the level of flexibility in these choices, as well as what foraging preferences enable certain pollinators and their host plants to thrive in their non‐native range. Research using DNA metabarcoding to investigate foraging behaviour of non‐native solitary bees has shown that pollinators are flexible in whether they forage on coevolved plants from their native range, or from their preferred plant families (Vaudo et al., 2020). DNA metabarcoding of pollen loads of honey bees and native insect pollinators in Australia found that their pollen diets overlap, and that the plant–pollinator network had a high level of generalization, which may lead to competition (Elliott et al., 2021). Other studies not directly focused on impacts of non‐native species have found that non‐native plant species occur more frequently in the diet of generalist bee species (Casanelles‐Abella et al., 2021; Wilson et al., 2021) and that increases in distributions of non‐native plant species have led to increased foraging on these species by honey bees (Jones, Brennan, et al., 2021). When additional studies are conducted in a broad range of ecosystems, we will begin to understand the context‐dependent impacts of non‐native species on plant–pollinator interactions. Thus, pollen DNA metabarcoding is likely to have an important role as a high‐throughput method enabling such broad ranging studies (Figure 2).

### 2.5 Monitoring and surveillance

A practical outcome of understanding the mechanisms behind the impacts of global ecological change on plants, pollinators and their interactions is the ability to monitor change as it occurs to study the dynamics of ecological communities, develop strategies to manage impacts of change and monitor the effectiveness of these management solutions. Community‐level monitoring of plant biodiversity through pollen DNA metabarcoding offers a high‐throughput alternative to botanical surveys (Johnson et al., 2021; Leontidou et al., 2021; Milla et al., 2022), and could be a valuable tool for the surveillance of ecosystem change, particularly when combined with other high‐throughput techniques such as remote sensing or unmanned aerial vehicle surveys (Ancin‐Murguzur et al., 2020). Important research questions investigating the effects of environmental disturbances and extreme climate events on community stability can be addressed with this monitoring approach. Monitoring activities will provide valuable data for understanding and predicting changes in plant and pollinator communities under environmental change. The large amounts of data generated by monitoring activities could be used to test hypotheses using environmental change as a natural experiment. For instance, long‐term monitoring of the same location under stress from anthropogenic impacts, or broad‐scale monitoring across heterogeneous landscapes could be used to assess how plant–pollinator networks restructure with changing resource availability (Figure 2).

Similar monitoring strategies are being used to monitor allergenic pollen. Pollen allergy, primarily causing allergic rhinitis, has significant impacts on human health. The seasonal presence of airborne pollen is linked strongly to plant phenology and therefore climate (Kurganskiy et al., 2021). Understanding the human health impacts of climate change related to pollen allergy requires an understanding of the relationship between plant phenology, seasonality and climate. The improved taxonomic resolution of pollen DNA metabarcoding can help address key questions about allergenic pollen, including: Does the length and timing of plant flowering period change according to preseasonal meterological conditions? Will future climate change lead to long‐term changes in the severity of the pollen season (Kurganskiy et al., 2021)? Addressing these questions will enable better forecasting of pollen season severity, with positive impacts on human health and quality of life. A major barrier to addressing these questions has been that plant families with highly allergenic pollen are difficult to identify through pollen morphology (e.g., Poaceae); however, the increased taxonomic resolution provided by DNA metabarcoding can distinguish allergenic species from nonallergenic species (e.g., Poaceae: Brennan et al., 2019; Urticaceae: Polling et al., 2022).

Airborne pollen sampling, combined with DNA metabarcoding, can also facilitate monitoring of allergenic species across large spatiotemporal scales (Brennan et al., 2019; Leontidou et al., 2018; Polling et al., 2022) and can identify seasonal changes in allergenic species (Campbell et al., 2020; Uetake et al., 2021). Better forecasting tools can now be developed to model how key allergenic species will respond under climate change (Kurganskiy et al., 2021). Finally, detailed modelling of allergenic pollen distribution would also be valuable for understanding plant–pollinator interactions under climate change. For example, this knowledge could be used to determine whether changes in plant phenology and distribution due to climate change could lead to a loss of pollinator availability and/or availability of novel pollinators, leading to the restructuring of ecological networks.

## 3 RESOLUTION OF TECHNICAL ISSUES AND ONGOING METHOD DEVELOPMENT

Pollen DNA metabarcoding is a relatively new method and is constantly improving. In this section, we highlight how some recent and ongoing method developments could enhance our ability to solve global change questions.

### 3.1 Quantification, sensitivity and detection probabilities

Many ecosystem changes initially present as changes in species abundances or interaction intensity rather than species composition, meaning that proportional or abundance data are much more informative than presence/absence. Furthermore, quantitative plant–pollinator networks have been found to reveal patterns that were not detectable with qualitative networks, such as asymmetrical dependencies between plants and pollinators (Bascompte et al., 2006), and may provide better predictions of secondary extinctions (Kaiser‐Bunbury et al., 2010). DNA metabarcoding is generally considered semiquantitative, and the proportion of reads can be considered a reasonable approximation of the proportion of species in a mixture (Bell et al., 2019; Polling et al., 2022). The quantitative accuracy of pollen DNA metabarcoding could be improved with a better understanding of species biases, method optimization and quantitative corrections. Imperfect quantification in DNA metabarcoding could result from biases among species at any step in the technical process (Lamb et al., 2019), or insufficient sequencing depth and/or replication (Deagle et al., 2019; Mata et al., 2019). Corrections for biases have been applied for other sample types (Garrido‐Sanz et al., 2022; Kembel et al., 2012; Lamb et al., 2019; Pawluczyk et al., 2015), and could be readily applied to pollen DNA metabarcoding. Amplification‐free methods eliminate the PCR (polymerase chain reaction) biases and have been shown in a handful of studies to be more quantitative than DNA metabarcoding (Bell et al., 2021; Lang et al., 2019; Peel et al., 2019). While existing methods help detect large‐scale changes in species proportions, the ongoing method development discussed here could lead to further improvements in quantification accuracy. This will enable improved understanding of ecosystem impacts through the more sensitive analyses that can be conducted with quantitative networks, and enable detection of responses that present as quantitative changes, rather than losses of species or links.

A related problem is understanding the sensitivity and expected detection limits for species of interest, and the rates of false positives and false negatives. This may be particularly relevant to ecosystem monitoring, where researchers may be interested in the presence or absence of low‐abundance species, such as a rare species becoming extinct or early detections of non‐native invasive species. These issues have been addressed in environmental DNA (eDNA) monitoring of water samples through site occupancy models which determine the confidence of presence/absence results based on species‐specific quantitative PCR assays (Dorazio & Erickson, 2018; Schmidt et al., 2013) and DNA metabarcoding (Ficetola et al., 2015). Similar methods would apply to pollen. Improved confidence in the presence of a species in a sample can be obtained by understanding the overall rate of false positives and false negatives for the study system and method. Researchers can increase confidence by using field‐ and laboratory‐based negative controls and positive controls or mock communities and no‐library negative controls to quantify sequencing mistag rates (Esling et al., 2015), and by ensuring adequate sequencing depth and replication (Shirazi et al., 2021).

Confidence estimates are also lacking for the classification steps in pollen DNA metabarcoding. There is an additional need to develop classification programs with more accurate probabilistic confidence estimates. While this has been attempted several times, available methods do not provide consistent results depending on the gene regions and databases used (Edgar, 2018).

### 3.2 Increasing taxonomic and genomic depth of reference databases

Having relatively complete reference databases, in terms of both species and gene regions, could increase the potential applications of pollen DNA metabarcoding. While it is possible to assess some ecological questions without identifying all taxa in the system, comparison between studies becomes difficult. Understanding the impacts of global change on plant–pollinator interactions will only be possible if the results of multiple studies can be compared over time and across regions. To do this requires fine‐scale taxonomic identification, which depends on a comprehensive reference library for the gene region being used for the species in the study system(s). There are an estimated 450,000 angiosperm species (Pimm & Joppa, 2015), and currently, around 25% of these have publicly available sequences for standard DNA barcodes (Bell et al., 2021). Reference libraries have been compiled for standard DNA barcodes for all flowering plants in the UK (Jones, Twyford, et al., 2021) and Canada (Kuzmina et al., 2017). The need to develop national databases has been recognized in other countries (Dormontt et al., 2018; Yang et al., 2020). Existing software such as bcdatabaser (Keller et al., 2020) or metacurator (Richardson et al., 2020) for creating custom databases from species lists can be helpful where there is no national database, different gene regions are being used or a more local database is desired. Several large‐scale projects are in progress to sequence DNA barcodes, organellar genomes and whole genomes for a large proportion of global biodiversity (Lewin et al., 2018). Careful archiving of raw data, amplicon sequence variants (ASVs) and/or representative sequences for operational taxonomic units (OTUs) will enable retrospective taxonomic classification once database gaps have been filled.

## 4 FUTURE RESEARCH DIRECTIONS

While current methodological constraints should be resolved, we also envisage several additional ways in which pollen metabarcoding could be developed to advance research on global change ecology.

### 4.1 Understanding the role of pollinators in plant population genetics

Pollen DNA metabarcoding currently works for species‐ or genus‐level identifications, but high‐throughput intraspecific identification is possible. Previous studies have demonstrated that intraspecific genetic variation can be analysed by sequencing single pollen grains (Hasegawa et al., 2009; Matsuki et al., 2007). With HTS, the pollen mixture on a pollinator could be analysed to reveal the genetic diversity of plant populations. Recent developments in the field of eDNA show some precedent for performing such population‐level analysis. For example, allele frequencies of both whale shark control region and nuclear microsatellites from round gobies matched estimates obtained from traditional methods (Andres et al., 2021; Sigsgaard et al., 2016). Thus, eDNA sampling of pollen might be an effective way of monitoring population‐level diversity of plants over time, without intensively collecting and sampling individuals. However, for high‐throughput intraspecific amplicon sequencing to succeed, more variable markers must be developed for plants because the standard DNA barcodes usually do not show much variation below the species level.

If both intra‐ and interspecific diversity could be examined from one pollen sample, micro‐ and macro‐evolutionary processes could be assessed at the level of individuals, species and communities. This combination of methods would enable new research on the role of pollinators in pollen‐mediated gene flow (Figure 2). The overall fitness and adaptive potential of plants within an area are closely related to the influx of diverse genes through pollen (Morente‐López et al., 2020). Studying the pollen on pollinators at an individual level (e.g., plant gametes) can uniquely show the diversity of genotypes that pollinators carry, rather than only the genotypes that are represented in the local plant community. Typically, the diversity of pollen transferred is assessed through genotyping parent plants and germinated offspring and applying parentage tests. Using an HTS approach on pollen could provide a more efficient way to study fundamental questions such as: Do changes in pollinator foraging behaviour affect outcrossing rates and fitness outcomes for plants? Do pollinators with larger foraging ranges carry a greater plant genetic diversity in their pollen load? Are the number of pollinators in an area proportional to the pollination service provided to plants?

Finally, combining novel intraspecific methods with ancient DNA technology could generate further research directions based on pollen DNA. Genomic DNA from Late Pleistocene bears, retrieved from shotgun sequencing, shows that it might be possible to use ancient eDNA for intraspecies analysis (Pedersen et al., 2021). This potentially opens not only the monitoring of genetic diversity of current populations through eDNA, but also the ability to compare trends to a historical record. Applying target capture of mitochondrial and nuclear DNA is a promising avenue of research for unlocking the potential stored in eDNA (Jensen et al., 2021). If these technologies were applied to pollen mixtures, pollinators might collect enough pollen to assess contemporary and historical trends in plant diversity over large areas without the need for labour‐intensive and expensive sampling of plant populations. This could provide tools to investigate how anthropogenic fragmentation has impacted plant genetic diversity, and whether allele frequencies in plant populations have changed over time in response to environmental changes.

### 4.2 Reconstructing ancient ecological change

Ecological change can be assessed through the comparison of contemporary ecological communities to past communities. Pollen preservation in ancient sediments, in combination with ancient sedimentary DNA (Capo et al., 2021), provides a resource for understanding past ecosystems. Usually, the pollen grains are examined morphologically, while the sediments are analysed through DNA sequencing to provide complementary data sources (Liu et al., 2021; Parducci et al., 2017). The ability to access the DNA inside pollen grains, for either single pollen grain analysis or DNA metabarcoding, would enable investigation of population dynamics in ancient ecosystems, something which is otherwise not possible in plants. This would improve the potential for using evolutionary approaches to understand ecosystem change. For example, the ancestry of populations could be traced by developing phylogenetic trees that include extinct and extant taxa as well as the direct comparison of ancient and extant sequences to establish direct links between extant and fossil samples in a species, providing genetic continuity through time. Pollen retrieved from lake sediments is an ideal material for ancient DNA analyses in plants because it is very abundant; depositional conditions are fast if the lake is not too deep (>10 m), reducing the degradation of pollen grains; pollen remains in situ once deposited in sediments; and there is a high degree of certainty to its stratigraphic context (Parducci et al., 2017; Parducci et al., 2019). By accessing the DNA in ancient pollen, there is great potential for characterizing past ecosystems (Niemeyer et al., 2017). Although it has been demonstrated that DNA is present in ancient pollen and can be sequenced (Bennett & Parducci, 2006; Parducci et al., 2005), ancient pollen samples are difficult to process, and there is a high risk of contamination with exogenous DNA. There are many different approaches for isolating and cleaning single pollen grains from the abundant pollen usually present in sediments. These include hand pipetting under a microscope, serial dilution, flow‐assisted cell sorting (flow cytometry), microfluidic manipulation (Wang & Navin, 2015), flow sorting or micromanipulation (Kron & Husband, 2012). Potential method development in this area could focus on improved efficiency and contamination control, and assessments to see if there are any biases due to DNA degradation over time. Finally, as we move into the future, it will be essential to retain and archive specimens for optimum preservation to be re‐analysed and compared to future samples, and nondestructive DNA extraction methods should be attempted.

### 4.3 Uptake of new sequencing technologies and adoption of minimum standards

Future advances in sequencing technology will enable researchers to answer questions that are currently logistically or technically difficult. New fast, portable sequencing technologies, such as Oxford Nanopore Technologies' MinION, could allow for analysis while in the field, enabling quicker results, management interventions, and removing the need for transport of biological material with its associated bureaucratic and sample preservation challenges. Use of long‐read technologies on pollen mixtures also has the potential to lead to improved understanding of pollinator preferences through more accurate quantification and increased resolution (Peel et al., 2019). Alternatively, the sequencing of restriction fragments (ddRAD [double digest restriction‐site associated DNA]) has been used to identify plant community composition from roots in soil samples (CAM et al., 2021), and the same method could easily be applied to pollen mixtures.

Pollen DNA metabarcoding represents an ideal method for comparative analysis across multiple study systems, to determine general mechanisms structuring communities of plants and pollinators. Pollen DNA metabarcoding could enable high‐quality global syntheses and meta‐analyses due to generation of compatible data, and its mandatory public deposition, accumulated between different workgroups across the globe and over extended periods over time. Such accumulated data sets can provide valuable insights into the differential effects of agriculture, urbanization and climatic change on pollination networks in comparisons between temperate and tropical regions, between continents and between different country conservation schemes. These types of studies would become more useful with adoption of minimum standards for replication, negative and positive controls, and method selection, as well as standardization of metadata reporting. The field of microbiomics is more advanced in this regard, with published codes of practice, standardization of reporting, and purpose‐built data repositories to increase the interpretability and comparison across studies (Dundore‐Arias et al., 2020; Field et al., 2008; Mukherjee et al., 2017; Yilmaz et al., 2011), and could serve as a guide for standardization of pollen DNA metabarcoding.

## 5 CONCLUSIONS

Plants, pollinators and their interactions are at risk from global ecological change, with implications for ecosystem services, economic productivity, and human health and quality of life (Figure 2). To contend with this, high‐throughput methods are essential for characterizing current biotic communities and monitoring how communities shift and decline with anthropogenic challenges. Pollen DNA metabarcoding and related methods are important tools that characterize plant and pollinator communities at a scale and resolution that was previously impossible. The global science community should invest in applying pollen metabarcoding to assemble a multiyear data set of plants, pollinators and their interactions across different habitat types, latitudes, elevations and levels of disturbances. Such a globally representative data set would improve our understanding of the mechanisms driving ecosystem change and provide evidence for real‐time management recommendations to preserve biodiversity and the evolutionary and ecological process that support it.

## AUTHOR CONTRIBUTIONS

Determining the scope of the paper: KLB (lead), in discussion with all authors. Compiling literature: KLB, FE‐V, AK, KN, RR, KT, AL, RML and NdV. Analysis of literature and creating figures and tables: KT. Writing first draft: KLB (lead), BB and KSB. Editing and reviewing the manuscript: all authors. All authors approved the final submitted manuscript.

## CONFLICT OF INTEREST

The authors declare no conflicts of interest.

## FUNDING INFORMATION

KLB was supported by internal funding from the University of Western Australia and CSIRO. KJT was supported by USDA‐NIFA grant no. 2021‐67012‐35153. RML was supported by the Biotechnology and Biological Sciences Research Council (BBSRC), part of UK Research and Innovation, through Core Strategic Programme Grant BB/CSP1720/1.

## Supporting information


Appendix S1

Appendix S2

## Data Availability

The data that support the findings of this study are available in the Supporting Information of this article. No biological samples were collected or analysed for this study.
