# Supplementary material for: Plants, pollinators and their interactions under global ecological change: The role of pollen DNA metabarcoding
Source: Mol Ecol. 2022 Sep 26;32(23):6345–62. doi: 10.1111/mec.16689 (PMC10947134; doi:10.1111/mec.16689)
Supplement: Supplementary file 1 — Appendix S1 [file MEC-32-6345-s002.pdf]

Supplemental Information for:

Plants, pollinators, and their interactions under global ecological change: the role of pollen DNA metabarcoding

Karen L. Bell, Katherine J. Turo, Abigail Lowe, Kevin Nota, Alexander Keller, Francisco Encinas-Viso, Laura Parducci, Rodney T. Richardson, Richard M. Leggett, Berry J. Brosi, Kevin S. Burgess, Yoshihisa Suyama, Natasha de Vere

Supplementary Methods

To assess the current application of pollen DNA metabarcoding to questions of global change, we completed a Web of Science search (accessed 11/30/2021) with the terms: “pollen” and “metabarcoding” or “pollen” and “meta-barcoding”. From a list of 134 results, we excluded irrelevant papers, reviews, and those that focused solely on methods development (Supplementary File S2). We also added several papers (n=28) to this list based on previous knowledge. Following these alterations, we examined a reduced list of 80 papers from 2014-2021 (Table S1). Generally, we found increasing numbers of papers with time, and a shift in 2017, from predominantly proof-of-concept papers to those exclusively focused on answering an ecological question of interest (Figure S1).

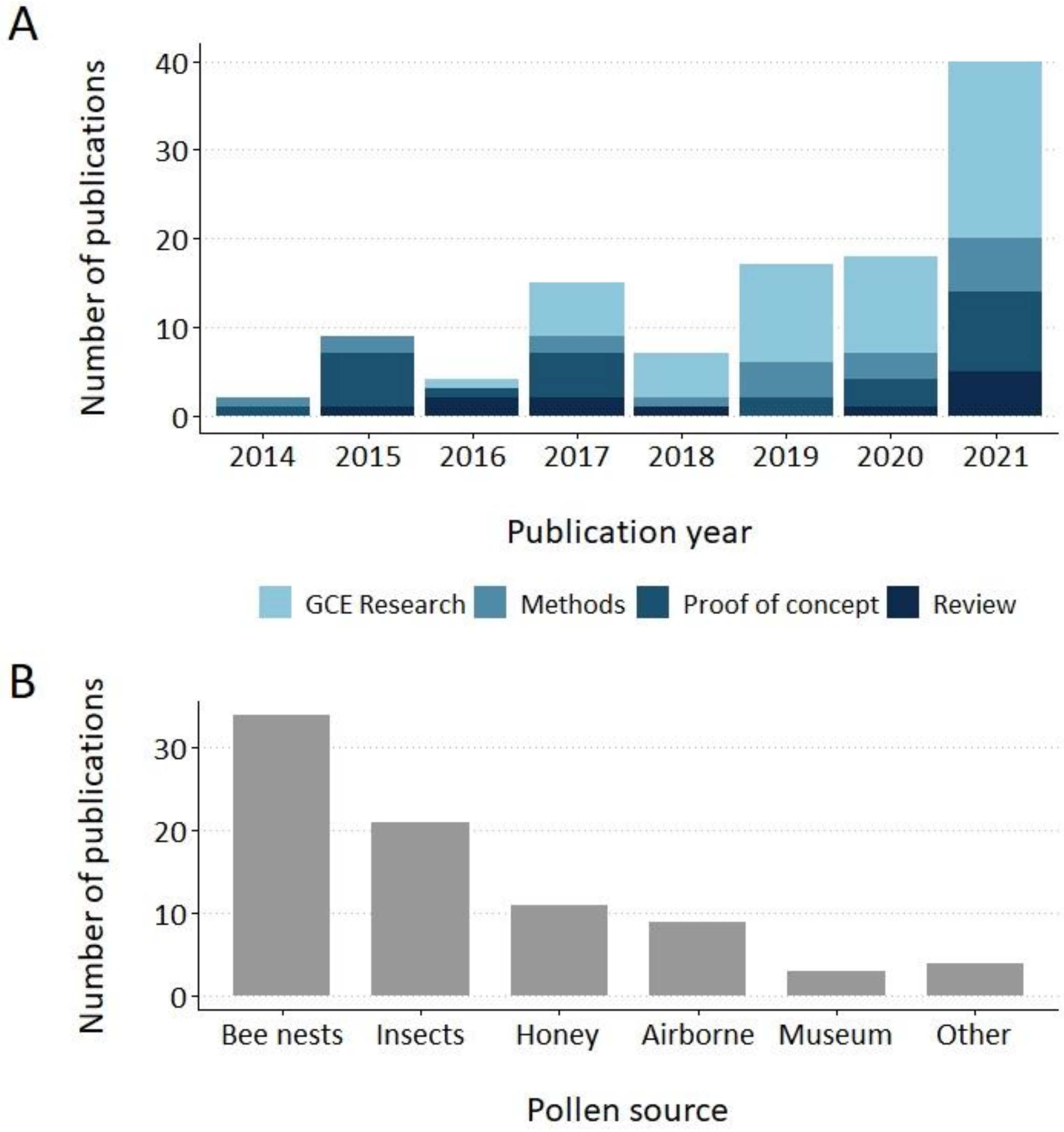

Figure S1. (A) Publications using or reviewing pollen DNA metabarcoding or related methods (n=112). Studies either apply pollen metabarcoding to questions in global change ecology, demonstrate proof-of-concept, develop metabarcoding methods, or review related literature. (B) Publications characterized by pollen source (n=80). Bee nests included pollen collected from honeybee hives with a pollen trap (n=20) and pollen provisions taken from solitary cavity nesting bees (n=15). Insects were predominately net or hand collected foraging bees (n=17) but some flies (Syrphidae) (n=7) and moths (n= 3) were also focal subjects. Pollen sourced from bats (n=1), a glacial core (n=1), and a commercially sourced pollen diet supplement (n=1) were categorized as Other.

MOLECULAR ECOLOGY

Table S1. Details of all papers from systematic review using pollen DNA metabarcoding to address an ecological hypothesis.

**Study system**	**Location**	**Research question**	**Sampling method**	**Gene Region**	**Reference database**	**Reference**
Derived honey bee products	Italy	What is the plant composition of a honey bee pollen pellet?	hive pollen trap	*rbcL*, *trnH-psbA*	NCBI GenBank	Galimberti et al. 2014, PLoS ONE
Derived honey bee products	Italy	Can DNA barcoding be used to identify plant species in multi-flower honey?	honey samples provided by beekeepers	*rbcL*, *trnH-psbA*	curated database from Grigna Settentrionale regional park	Bruni et al. 2015, Food Chemistry
Honey bee foraging	USA	Can pollen metabarcoding characterize honey bee foraging?	hive pollen trap	ITS1, ITS2	NCBI GenBank	Cornman et al. 2015, PLoS ONE
Honey bee foraging	UK	What is the floral composition of honey?	honey samples provided by beekeepers	*rbcL*	curated database from Barcode Wales, NCBI GenBank	Hawkins et al. 2015, PLoS ONE
Honey and solitary bee foraging	Germany	Can pollen metabarcoding reveal bee foraging from a mixed pollen sample?	hive pollen trap, cotton swab of *Osmia bicornis* nesting cell walls	ITS2	ITS2 database curated for Bavarian flora, NCBI GenBank	Keller et al. 2015, Plant Biology
Airborne pollen	Netherlands	Can metabarcoding quantify pollen from airborne samples at high taxonomic resolution?	Burkard volumetric air sampler with adhesive tape	*trnL*	NCBI GenBank	Kraaijeveld et al. 2015, Molecular Ecology Resources
Honey bee foraging	USA	How do metabarcoding and microscopy differently characterize honey bee foraging in an agroecosystem?	hive pollen trap	ITS2	NCBI GenBank, curated with occurrences in USDA PLANTS	Richardson et al. 2015, Applications in Plant Sciences
Pollinator foraging	USA	Does floral diversity predict microbiome diversity in a pollen provision?	pollen provisions from cavity nest	*rbcL*	NCBI GenBank, curated with occurrences in USDA PLANTS	McFrederick and Rehan 2016, Molecular Ecology

Pollinator foraging	France	Can metabarcoding reveal and quantify plant pollinator interactions better than observational data?	netted insect pollinators (bees, flies, beetles)	*trnL*, ITS1	EMBL database (Ficetola at al. 2010, BMC Genomics)	Pornon 2016, Scientific Reports
Pollinator foraging	USA	Can pollen metabarcoding reconstruct plant-pollinator networks?	netted bee individuals	ITS2, *rbcL*	NCBI GenBank	Bell et al. 2017, Applications in Plant Sciences
Honey bee foraging	Germany	How does pollen foraging of honey bee colonies change in response to agricultural landscape structure and seasonality?	hive pollen trap	ITS2	curated from Bavarian plant list	Danner et al. 2017, PLoS ONE
Honey bee foraging	UK	How can honey reveal honey bees' use of nectar and pollen resources?	fresh honey collected from comb	*rbcL*	curated from Barcode Wales project	De Vere et al. 2017, Scientific Reports
Honey bee foraging	UK	Is the pollen diversity of bee bread linked to its nutrient content?	hive pollen trap	ITS2	NCBI GenBank, Viridiplantae ITS2 Database	Donkersley et al. 2017, Oecologia
Pollinator foraging	France	What is the complete plant-pollinator foraging network in a semi-natural montane grassland?	hand-collected insect pollinators (bees, flies)	ITS2	ITS2 database, ITS plant rDNA cistron sequences from NCBI GenBank	Galliot et al. 2017, Journal of Insect Conservation
Social bee foraging	Australia	To what extent do bees use diverse forage in species-rich and resource-abundant environments?	corbicular loads from netted *Tetragonula carbonaria*	ITS2	NCBI GenBank (Sickel et al. 2015)	Kaluza et al. 2017, Ecosphere
Airborne pollen	Finland	Are there seasonal changes in indoor airborne pollen diversity?	vacuuming dust from surfaces	ITS2	NCBI GenBank	Korpelainen and Pietiläinen 2017, Nordic Journal of Botany
Derived honey bee products	India	Can multi-locus metabarcoding characterize the floral composition of honey?	honey samples provided by beekeepers	*matK*, *rbcL*, ITS2	NCBI Viridiplantae database	Laha et al. 2017, AMB Express

Honey bee foraging	USA	What are the seasonal patterns in colony recruitment dancing for pollen?	hand-collected honey bees	*matK*, *rbcLa*	NCBI GenBank curated with San Diego County plant occurances	Park and Nieh 2017, Insectes Sociaux
Pollinator foraging	France	How do the properties of pollination networks change with data collected from DNA metabarcoding?	netted insect pollinators (bees, flies, beetles)	*trnL*, ITS1	EMBL database (Ficetola at al. 2010, BMC Genomics)	Pornon et al. 2017, Scientific Reports
Honey bee foraging	USA	How do metabarcoding and microscopy differently characterize honey bee foraging in high and low intensity agricultural settings?	hive pollen trap	ITS1, ITS2	NCBI GenBank	Smart et al. 2017, Environmental Entomology
Lepidopteran migration	China	What is the spatiotemporal origin of a noctuid moth that migrates long distances?	vertical-pointing searchlight trap	*rbcL*	NCBI GenBank	Chang et al. 2018, International Journal of Molecular Sciences
Solitary bee foraging	UK	Do cavity nesting bees forage on wildflowers planted in agri-environment schemes?	pollen provisions from cavity nest	ITS2	curated database from Royal Botanic Gardens, Kew and collected reference samples	Gresty et al. 2018, Ecology and Evolution
Honey bee foraging	Japan	What plants do honey bees forage on?	hive pollen trap	*trnL*, ITS2	NCBI GenBank	Kamo et al. 2018, Applied Entomology and Zoology
Syrphidae foraging	UK	What is the structure of hoverfly pollen transport networks?	netted hoverfly individuals	*rbcL*	curated database	Lucas et al. 2018, Journal of Animal Ecology
Syrphidae foraging	UK	Do hoverfly genera exhibit functional complementarity in plant-pollinator networks?	netted hoverfly individuals	*rbcL*	curated database	Lucas et al. 2018, Scientific Reports

Pollinator foraging	Czech Republic	How does bumble bee workforce reduction alter pollen foraging?	pollen provisions from colony	ITS2	plant reference specimens, curated NCBI GenBank	Biella et al. 2019, PLoS ONE
Airborne pollen	UK	How does the taxonomic composition of grass pollen change throughout the season?	Burkard volumetric air sampler with adhesive tape	ITS2, *rbcL*	curated database, NCBI GenBank	Brennan et al. 2019, Nature Ecology and Evolution
Historic foraging reconstruction	South Africa	Can museum specimens reveal plant-pollinator interactions across time?	micropipette tip dipped in glycerol on bee abdomen	ITS1, ITS2, *rbcL*	NCBI GenBank	Gous et al. 2019, Evolutionary Applications
Honey bee foraging	Switzerland	Can pollen metabarcoding of honey be used to assess honey bee foraging across an urbanization gradient?	honey samples provided by beekeepers	ITS2	NCBI GenBank	Lucek et al. 2019, Apidologie
Nocturnal moth foraging	UK	What different plant-pollinator interactions do microscopy and metabarcoding reveal for nocturnal moths?	moth proboscis	*rbcL*	NCBI GenBank	McGregor 2019, Ecological Entomology
Pollinator foraging	Australia	How does pollen usage and nest microbial communities change across landscapes?	pollen provisions from cavity nest	*rbcL*	NCBI GenBank	McFrederick and Rehan 2019, Microbial Ecology
Pollinator foraging	Germany	How do honey bee waggle dances increase the diversity of pollen diets in intensively managed agricultural landscapes?	hive pollen trap	ITS2	ITS2 database v. 5, NCBI GenBank curated for German plants	Nürnberger et al. 2019, Molecular Ecology
Pollinator foraging	France	How do native mass-flowering plants affect the specialization of insects at individual and species levels?	netted insect pollinators (bees, flies, beetles)	*trnL*, ITS1	EMBL database (Ficetola at al. 2010, BMC Genomics)	Pornon et al. 2019, Ecology and Evolution
Urban bee foraging	UK	How do urban bees use sown wildflower strips?	netted bee individuals	*rbcL*	curated database from Barcode Wales, NCBI GenBank	Potter et al. 2019, PeerJ

Honey bee foraging	USA	What is the phenological progression of honey bee spring foraging in an agroecosystem?	hive pollen trap	*rbcL, trnL, trnH* and ITS2	NCBI GenBank, Viridiplantae ITS2 Database	Richardson et al. 2019, Molecular Ecology
Lepidopteran migration	Spain	Can pollen metabarcoding track long-distance insect migrations?	netted butterfly individuals	ITS2	Viridiplantae database, NCBI GenBank	Suchan et al. 2019, Molecular Ecology Resources
Biomonitoring	Canada	Can honey bee hives be used to biomonitor plant pests?	hive pollen trap	ITS2	NCBI GenBank	Tremblay et al. 2019, Environmental DNA
Pollinator foraging	Germany	How does pollen collection influence bacterial communities in bee nests?	pollen provisions from cavity nest	ITS2	ITS2 database curated for Bavarian flora, NCBI GenBank	Voulgari-Kokota et al. 2019, Ecology and Evolution
Airborne pollen	Italy	Can DNA metabarcoding be used to simultaneously analyze airborne fungal and plant particles?	volumetric air sampler with adhesive tape	ITS2	PLANiTS database curated from NCBI GenBank	Banchi et al. 2020, Science of the Total Environment
Social and honey bee foraging	Germany	How do bees' diets shift along a gradient of mass flowering crops?	hive pollen trap, netted bumble bees	ITS2	magicBLAST database	Bänsch et al. 2020, Molecular Ecology
Airborne pollen	Australia	What is the utility of pollen metabarcoding for tracking seasonal changes in pollen allergens?	Burkard volumetric air sampler with adhesive tape	*rbcL*	curated database Rocklea region, NCBI GenBank	Campbell et al. 2020, Science of the Total Environment
Pollinator foraging	USA	Do the pollen and microbial components of a bee's diet change across its range?	pollen provisions from cavity nest	*rbcL*	NCBI GenBank, curated with occurrences in USDA PLANTS	Dew et al. 2020, Insects
Derived honey bee products	Iran	Can pollen metabarcoding be used to determine honey's geographical origin?	honey samples provided by beekeepers	ITS2, *rbcL*	NCBI GenBank	Khansaritoreh et al. 2020, Heliyon

Pollinator foraging	Austria	How does land use affect *Osmia cornuta*'s pollen foraging?	pollen provisions from cavity nest	*trnL*	NCBI GenBank	Kratschmer et al. 2020, Ecological Entomology
Honey bee foraging	USA	Do honey bees use pollen provided by Conservation Reserve Program (CRP) pollinator plantings?	hive pollen trap	*rbcL, trnL*, ITS2	NCBI GenBank curated with USDA plant occurrences	McMinn-Sauder et al. 2020, Insects
Pollinator foraging	USA	Does landscape structure influence bee diet (pollen diversity) and macronutrient gut content in managed and native bees?	pan trapped bees, alimentary canal dissected	*trnL*	NCBI GenBank	Mogren et al. 2020, Conservation Physiology
Honey bee foraging	USA	Can honey bee foraging reveal the overall availability and taxonomic composition of floral resources in an urban environment?	hive pollen trap	*trnL*, ITS1, ITS2	NCBI GenBank curated with USDA plant occurrences	Sponsler et al. 2020, Ecosphere
Honey bee foraging	USA	Which ornamental plants do honey bees forage on?	hive pollen trap	ITS2	NCBI GenBank curated with USDA plant occurrences	Sponsler et al. 2020, Ecosphere
Honey bee foraging	Japan	Which urban plants are pollen and nectar sources for honey bees?	hive pollen trap	*rbcL*	NCBI GenBank	Tanaka et al. 2020, BMC Research Notes
Social bee foraging	Australia	Does plant species richness correlate with floral diversity and nutritional quality of larval provisions?	corbicular loads from netted *Tetragonula carbonaria*	ITS2	global ITS2 reference database created with 'bcdatabaser'	Trinkl et al. 2020, Insects
Pollinator foraging	USA	Does plant origin or taxa influence *Osmia cornifron*'s pollen foraging?	pollen provisions from cavity nest	ITS2	NCBI GenBank curated with USDA plant occurrences	Vaudo et al. 2020, Royal Society Open Science
Pollinator foraging	USA	How does characterization of plant-pollinator foraging networks and bee's diet breadth change when using observational data vs. pollen DNA?	netted bee individuals	ITS2, *rbcL*	NCBI GenBank curated with regional plant list and reference specimens	Arstingstall et al. 2021, Molecular Ecology

MOLECULAR ECOLOGY

Bat foraging	Australia	Can bat diets be quantified with pollen DNA metabarcoding methods?	bat fecal samples	ITS2, *rbcL*	NCBI GenBank	Bell et al. 2021, Australian Journal of Zoology
Social bee foraging	Estonia	What are the foraging preferences of bumblebees in a heterogeneous landscape?	trap attached to commercial bumble bee hive	ITS2	NCBI GenBank curated for Estonian plants	Bontsutsnaja et al. 2021, Insects
Solitary bee foraging	Belgium, France, Estonia, Poland, Switzerland	Does urbanisation affect larval diet and solitary bee species distribution?	pollen provisions from cavity nest	ITS2	ITS2 reference database generated with BCdatabaser (Keller et al., 2020, Bioinformatics)	Casanelles-Abella et al. 2021, Journal of Applied Ecology
Derived honey bee products	Italy	Can pollen DNA metabarcoding identify honey origin?	honey samples provided by beekeepers	*trnL*	NCBI GenBank	Chiara et al. 2021, Food Control
Solitary bee foraging	Belgium	Do mass-flowering crops affect reproduction of wild solitary bees?	pollen provisions from cavity nest	ITS2	NCBI GenBank	Eeraerts et al. 2021, Basic and Applied Ecology
Pollinator foraging	Australia	What is the overlap in resource use between native bee species and managed and feral honeybees?	netted bee individuals	*rbcL*	curated reference database of heathland species	Elliott et al. 2021, Basic and Applied Ecology
Historic foraging reconstruction	South Africa	What are the host plants of leaf-cutter bees, and do they vary among biomes for widespread bee species?	micropipette tip scraping bee abdomen	ITS2	NCBI GenBank, Viridiplantae ITS2 Database	Gous et al. 2021, PLoS ONE
Derived honey bee products	Italy	Can metabarcoding determine the floral origin of fungi and pyrrolizidine alkaloids in pollen?	pollen samples provided by beekeepers, hive pollen trap	ITS2	ITS2 Viridiplantae sequences from PLANiTS (Banchi et al., 2020)	Inacio et al. 2021, Food Research International

Pollinator foraging	USA	Are observations of plant pollinator-contact effective representation of pollen carriage? How does this as a scientific method compare to two methods of pollen DNA barcoding?	netted bee individuals	curated gene region for *Clarkia* spp.	curated database of 4 *Clarkia* spp.	James et al. 2021, Molecular Ecology Resources
Derived honey bee products	UK	How have changes in the landscape affected honey bee foraging?	honey samples provided by beekeepers	ITS2, *rbcL*	curated from Barcode Wales project	Jones et al. 2021, Communications Biology
Airborne pollen	USA	Can pollen DNA metabarcoding forensically determine geographic provenance of a settle dust sample?	passive particle settling on glass microscope slides	ITS2, *rbcL*	NCBI GenBank	Lennartz et al. 2021, Scientific Reports
Airborne pollen	Italy	What is the usefulness of pollen DNA metabarcoding to assess plant biodiversity from airborne pollen samples?	Tauber trap	*trnL*	NCBI GenBank	Leontidou et al. 2021, Scientific Reports
Solitary bee foraging	China	What are the forage plants of *Osmia excavata*, and how does this vary with land use and region?	pollen provisions from cavity nest	ITS2	NCBI GenBank	Lu et al. 2021, Frontiers in Ecology and Evolution
Solitary bee foraging	Tanzania	How does an elevational gradient affect cryptic speciation and pollen foraging in *Lasioglossum* species?	bee individuals collected with sterile jars	ITS2	NCBI GenBank	Mayr et al. 2021, Ecology and Evolution
Pollinator foraging	France	How do plant-pollinator networks vary with climate, altitude, landscape and management?	bee individuals collected with Falcon tubes	ITS2	NCBI GenBank	Michelot-Antalik et al. 2021,
Derived honey bee products	Australia	What plants do honey bees forage on in different regions?	honey samples provided by beekeepers	ITS2, *trnL*	ITS2DB (Ankenbrand et al., 2015) and PlantAligDB trnL_GH (Santos et al., 2019) databases curated with occurrence	Milla et al. 2021, Ecology and Evolution

MOLECULAR ECOLOGY

					records from Atlas of Living Australia	
Social bee foraging	Germany	Are flower fields effective for providing foraging habitat for bumblebees?	bee individuals caught in vial, washed with water, and released	ITS2	ITS2 reference database generated with BCdatabaser (Keller et al., 2020, Bioinformatics)	Piko et al. 2021, Basic and Applied Ecology
Solitary bee foraging	Belgium	Does increased resource availability dilute parasite transmission?	pollen provisions from cavity nest	ITS2	NCBI GenBank	Piot et al. 2021, International Journal of Parasitology
Honey bee foraging	USA	How does land use and season affect pollen foraging and protein content?	hive pollen trap	ITS1, ITS2	NCBI GenBank curated with occurrences in USDA PLANTS	Quinlan et al. 2021, Agriculture, Ecosystems & Environment
Honey bee foraging	USA	How does land use affect species diversity and temporal turnover of plants foraged by honey bees?	hive pollen trap	*rbcL, trnL* and ITS2	NCBI GenBank curated with occurrences in USDA PLANTS	Richardson et al. 2021, Molecular Ecology
Airborne pollen	UK	How does airborne pollen of different grass species vary with space and time, and which species are responsible for allergies?	Burkard volumetric air sampler with 1.5 ml microcentrifuge tube	ITS2	curated database, NCBI GenBank	Rowney et al. 2021, Current Biology
Historic foraging reconstruction	USA	What are the host plants of endangered *Bombus affinus*, and have they changed over time?	Corbicular loads from museum specimens	ITS2	NCBI GenBank curated with occurrences in USDA PLANTS	Simanonok et al. 2021, Biodiversity and Conservation

Pollinator foraging	Tanzania	How are plant and pollinator insect assemblages and interactions in Sub-Saharan farming conditions are shaped by land use intensification?	netted bee individuals	ITS2	plant reference specimens, curated NCBI GenBank	Tommasi et al. 2021, Agriculture, Ecosystems & Environment
Airborne pollen	Japan	What species are in the airborne pollen and how does this vary seasonally?	sequential aerosol sampler	*rbcL*	*rbcL* database (Bell et al., 2017)	Uetake et al. 2021, Science of the Total Environment
Glacial core	Italy	Can pollen metabarcoding estimate historical plant biodiversity archived in glacier cores?	glacial core	*trnL*	NCBI GenBank	Varotto et al. 2021, Scientific Reports
Social bee foraging	Australia	What pollen resources are used by a native social bee?	pollen provisions from colony	ITS2, *rbcL*	NCBI GenBank curated by plant occurrence at study site	Wilson et al. 2021, Agriculture, Ecosystems & Environment
Derived honey bee products	Sweden, Finland, Estonia	Can pollen, bacterial or fungal DNA isolate the geographic region of honey? Which data performs best?	honey samples provided by beekeepers	ITS2, *rbcL*a and *trnL*	NCBI GenBank curated for occurrence in Scandinavia	Wirta et al. 2021, Scientific Reports
Derived honey bee products	UK	Are accumulated neonicotinoids present in honey, and does this vary with foraged plant species?	honey samples provided by beekeepers	ITS2	NCBI GenBank	Woodcock et al. 2021, Agriculture, Ecosystems & Environment
